# The Role of Premorbid IQ and Age of Onset as Useful Predictors of Clinical, Functional Outcomes, and Recovery of Individuals with a First Episode of Psychosis

**DOI:** 10.3390/jcm10112474

**Published:** 2021-06-02

**Authors:** Mariola Molina-García, David Fraguas, Ángel del Rey-Mejías, Gisela Mezquida, Ana M. Sánchez-Torres, Silvia Amoretti, Antonio Lobo, Ana González-Pinto, Álvaro Andreu-Bernabeu, Iluminada Corripio, Eduard Vieta, Inmaculada Baeza, Anna Mané, Manuel Cuesta, Elena de la Serna, Beatriz Payá, Iñaki Zorrilla, Celso Arango, Miquel Bernardo, Marta Rapado-Castro, Mara Parellada, on behalf of the PEPs Group

**Affiliations:** 1 Department of Child and Adolescent Psychiatry, Institute of Psychiatry and Mental Health, Gregorio Marañón Health Research Institute (IiSGM), Hospital General Universitario Gregorio Marañón, Research Networking Center for Mental Health Network (CIBERSAM), School of Medicine, Complutense University of Madrid (UCM), 28007 Madrid, Spain; alvaro.andreu@iisgm.com (Á.A.-B.); carango@hggm.es (C.A.); mrapado@iisgm.com (M.R.-C.); parelladahggm@gmail.com (M.P.); 2 Institute of Psychiatry and Mental Health, Hospital Clínico San Carlos (IdISSC), Research Networking Center for Mental Health Network (CIBERSAM), School of Medicine, Complutense University of Madrid (UCM), 28040 Madrid, Spain; david.fraguas@iisgm.com; 3 Data Science Unit, Instituto Ramón y Cajal de Investigación Sanitaria (IRYCIS), Hospital Universitario Ramón y Cajal, School of Psychology, Complutense University of Madrid (UCM), 28223 Madrid, Spain; alreymejias@gmail.com; 4 Barcelona Clinic Schizophrenia Unit, Neuroscience Institute, August Pi I Sunyer Biomedical Research Institute (IDIBAPS), Hospital Clinic of Barcelona, Research Networking Center for Mental Health Network (CIBERSAM), University of Barcelona, 08036 Barcelona, Spain; MEZQUIDA@clinic.cat (G.M.); AMORETTI@clinic.cat (S.A.); bernardo@clinic.cat (M.B.); 5 Department of Psychiatry, Navarra Institute for Health Research (IdiSNA), Complejo Hospitalario de Navarra, 31008 Pamplona, Spain; am.sanchez.torres@navarra.es (A.M.S.-T.); mj.cuesta.zorita@navarra.es (M.C.); 6 Department of Medicine and Psychiatry, Instituto de Investigación Sanitaria Aragón (IIS Aragón), Universidad de Zaragoza, 50009 Zaragoza, Spain; alobosat@gmail.com; 7 Department of Psychiatry, Bioaraba Health Research Institute, Hospital Universitario de Álava, Research Networking Center for Mental Health Network (CIBERSAM), University of the Basque Country (UPV/EHU), 01009 Vitoria, Spain; anapinto@telefonica.net (A.G.-P.); inaki.zorrillamartinez@osakidetza.eus (I.Z.); 8 Department of Psychiatry, Institut d’Investigació Biomèdica-Sant Pau (IIB-SANT PAU), Hospital de la Santa Creu i Sant Pau, Research Networking Center for Mental Health Network (CIBERSAM), Universitat Autònoma de Barcelona (UAB), 08041 Barcelona, Spain; ICorripio@santpau.cat; 9 August Pi i Sunyer Biomedical Research Institute (IDIBAPS), Institute of Neurosciences, Hospital Clinic, Research Networking Center for Mental Health Network (CIBERSAM), University of Barcelona, 08036 Barcelona, Spain; evieta@clinic.cat; 10 Child and Adolescent Psychiatry and Psychology Department, SGR-881, August Pi i Sunyer Biomedical Research Institute (IDIBAPS), Hospital Clínic de Barcelona, Research Networking Center for Mental Health Network (CIBERSAM), Universitat de Barcelona, 08036 Barcelona, Spain; IBAEZA@clinic.cat; 11 Hospital del Mar Medical Research Institute, Research Networking Center for Mental Health Network (CIBERSAM), Autonomous University of Barcelona, 08003 Barcelona, Spain; manesantacana@yahoo.es; 12 Child and Adolescent Psychiatry and Psychology Department, Hospital Clinic of Barcelona, Research Networking Center for Mental Health Network (CIBERSAM), 08036 Barcelona, Spain; ESERNA@clinic.cat; 13 IDIVAL, Hospital Universitario Marqués de Valdecilla, 39008 Santander, Spain; mbeatriz.paya@scsalud.es; 14 Melbourne Neuropsychiatry Centre, The University of Melbourne and Melbourne Health, 161 Barry Street, Carlton South, VIC 3053, Australia

**Keywords:** psychosis, first-episode, premorbid intelligence, age at onset, functional outcome, remission, recovery, heterogeneity, subgroup

## Abstract

Background: premorbid IQ (pIQ) and age of onset are predictors of clinical severity and long-term functioning after a first episode of psychosis. However, the additive influence of these variables on clinical, functional, and recovery rates outcomes is largely unknown. Methods: we characterized 255 individuals who have experienced a first episode of psychosis in four a priori defined subgroups based on pIQ (low pIQ < 85; average pIQ ≥ 85) and age of onset (early onset < 18 years; adult onset ≥ 18 years). We conducted clinical and functional assessments at baseline and at two-year follow-up. We calculated symptom remission and recovery rates using the Positive and Negative Symptoms of Schizophrenia Schedule (PANSS) and the Global Assessment Functioning (GAF or Children-GAF). We examined clinical and functional changes with pair-wise comparisons and two-way mixed ANOVA. We built hierarchical lineal and logistic regression models to estimate the predictive value of the independent variables over functioning or recovery rates. Results: early-onset patients had more severe positive symptoms and poorer functioning than adult-onset patients. At two-year follow-up, only early-onset with low pIQ and adult-onset with average pIQ subgroups differed consistently, with the former having more negative symptoms (*d* = 0.59), poorer functioning (*d* = 0.82), lower remission (61% vs. 81.1%), and clinical recovery (34.1% vs. 62.2%). Conclusions: early-onset individuals with low pIQ may present persistent negative symptoms, lower functioning, and less recovery likelihood at two-year follow-up. Intensive cognitive and functional programs for these individuals merit testing to improve long-term recovery rates in this subgroup.

## 1. Introduction

There is large variability in clinical presentation, course of symptoms [1,2], and subsequent clinical and/or functional deterioration [3,4,5,6,7] among first episode of psychosis individuals. According to the literature, about 50% to 78% of individuals who have experienced a first episode of psychosis achieve symptomatic remission following the comprehensive treatment by early-intervention services and antipsychotic treatment [8,9,10]. Good functional outcome is rarer, with the highest rates as high as 51% [11,12,13,14]. Recovery, a concept which implies both symptom remission and good functioning [15], occurs even less frequently (around 40% [16]). Given the variability, the study of predictors of recovery in first episode of psychosis is essential to identify and characterize patients at higher risk of poor long-term functioning. Two of the most common predictors of clinical and functional outcomes in individuals with psychosis, both in clinical practice and research, are the age at onset of psychotic symptoms [17,18,19,20,21] and premorbid intellectual performance (pIQ) [22,23,24,25,26]. These aspects are remarkably variable: the onset of psychosis ranges from childhood to adulthood [27], and premorbid cognitive function ranges from severe impairment to average performance [28].

Regarding age of onset, studies directly comparing individuals with an early age of onset of psychotic symptoms (before the age of 18) with individuals with adult-onset (18 years and older) have concluded that the early-onset subgroup is more likely to display worse premorbid functioning [29], higher primary negative symptoms [27,30], later positive symptom remission [21], and worse functional prognosis [31,32,33]. On the other hand, regarding the wide variability in premorbid intellectual performance among individuals with psychosis who develop schizophrenia [25,34], meta-analitic evidence has held the important role of low premorbid intellectual performance in terms of functional prognosis in psychotic disorders [35,36,37]. It has been estimated that among individuals with a psychotic disorder the risk of schizophrenia diagnosis increases 3.7% by every one point of lower pIQ, being greater for participants with early-onset who present with a pIQ between 70 and 85 [23]. Previous work on the relationship between age of onset of psychotic symptoms and pIQ [25,28] suggested that the presence of a lower pIQ was a risk factor for an earlier onset and worse functioning [28]. Furthermore, recent evidence signals that among individuals with a first episode of psychosis, pIQ lower than 85 correlates with an almost four times higher probability of EO [38]. In addition, evidence supports the idea that early onset of schizophrenia is moderately heritable [39,40], and that there is a familial component in the relationship between cognitive performance and psychosis [41,42]. Thus, the presence of an early age of onset and a low premorbid pIQ may reflect a different neurodevelopmental course of psychotic disorder [43].

Even though acummulated evidence suggests that both low pIQ and early age of onset could help explain recovery rates of individuals who have experienced a first episode of psychosis, there is less evidence with regards to the additive influence of these two variables on clinical symptomatology and psychosocial functioning. Previous work has compared subgroups of patients with psychotic disorders based on their age of onset or premorbid IQ, but the additive impact of these two factors over clinical and functioning outcomes has not been explored by a subgroup strategy. The present two-year follow-up study aims to bridge this research gap by means of a subgroup analysis strategy. We explored a large group of individuals with a first episode of psychosis divided into four a priori specified subgroups based on age onset, before or after 18 years old, and pIQ, below or above 85, describing and comparing clinical and functioning outcomes from the resulting subgroups at baseline and at two-year follow-up. We hypothesized that the subgroup of participants with an early age of onset and lower pIQ would show higher symptom scores and worse general functioning both at baseline and at two-year follow-up, in comparison with other first episode of psychosis subgroups with higher age of onset and higher pIQ. In addition, we hypothesized that the subgroup of early-onset with low pIQ would present with the lowest percentage rate of symptom remission and clinical recovery compared to the other subgroups of patients with a first episode of psychosis at the two-year follow-up assessment.

## 2. Methods

### 2.1. Participants

The present study included a total of 255 individuals who have experienced a first episode of psychosis (age range 10–36 years) who participated in two longitudinal, multicenter studies in Spain with shared methodology: the “Children and adolescent first episode psychosis study (CAFEPS study)” [44], for individuals up to 18 years old with a first episode of psychosis, and the “Phenotype-genotype and environmental interaction; application of a predictive model in first psychotic episodes (PEPs study)” for subjects between 7 and 35 years with a first episode of psychosis [45,46,47]. The two study cohorts underwent the same clinical and neuropsychological evaluations. A detailed description of the methodologies is available in Bernardo et al. [45] and Castro-Fornieles et al. [44]. In brief, the CAFEPS study team recruited 110 children and adolescents with a first episode of psychosis from six clinical centers, from March 2003 to November 2005. In addition, the PEPs study [47] included 335 subjects with a first episode of psychosis from 16 clinical centers from April 2009 to April 2011. All patients and/or their parents or legal guardians provided written informed consent for inclusion before they participated in the study. Both studies were conducted in accordance with the Declaration of Helsinki, and the protocols were approved by the Ethics Committee of each participating clinical center (project identification code CAFEPS: G03032; and PEPs: 2008/4232). For this study, we included only those participants who had completed evaluation of estimated premorbid IQ, as well as functional and clinical assessments both at baseline and at two-year follow-up. As a result, we analyzed data for a subsample of a total 255 individuals with a first episode of psychosis (79 of them derived from the CAFEPS, and 176 from the PEPs study).

Participants had to meet the following inclusion criteria: (1) age between 7 and 17 years at the time of first evaluation in the CAFEPS study and between 7 and 35 years in the PEPs study; (2) presence of positive psychotic symptoms, such as delusions or hallucinations, lasting less than 12 months within the context of a first episode of psychosis; (3) participants spoke and understood Spanish; and (4) gave informed consent. Exclusion criteria for participants were: (1) the presence of any other Axis I disorder at the time of evaluation that might account for the psychotic symptoms (such as substance abuse, autistic spectrum disorders, post-traumatic stress disorder, or acute stress disorder); (2) intellectual disability as per DSM-IV criteria (including not only an IQ below 70 but also impaired functioning or pervasive developmental disorder); (3) presence of neurological disorders, and organic disease with mental repercussions; (4) history of head trauma with loss of consciousness; and (5) pregnancy. Occasional substance use was not an exclusion criterion if positive symptoms persisted for more than two weeks after a negative urine drug test.

### 2.2. Procedures

#### 2.2.1. Definition of Premorbid IQ and Age of Onset Subgroups

As a measure of premorbid IQ we used the Vocabulary subtest of the Spanish versions of the Wechsler Intelligence Tests: Wechsler Children Intelligence Scale WISC-R [48] or WISC-IV [49] for individuals aged ≤16 and the Wechsler Adult Intelligence Scale, WAIS-III for individuals aged ≥17 [50]. The Vocabulary subtest of the Wechsler tests has been previously used as a proxy measure for premorbid intelligence in samples of first episode of psychosis patients [51]. It has been shown that the vocabulary task is relatively unaffected by psychopathology [52] and by neurodegenerative processes, such as dementia [53]. It is a task based on an individual’s general knowledge of linguistic information (phonology and semantics), which has been generally associated with crystallized intelligence [54,55], provided that the participants are evaluated in their mother tongue. We used standardized scores of vocabulary subtest of WAIS-III or WISC-R/IV to compute the estimated score following the formula proposed by Lyman Howard [56] (i.e., vocabulary standardized score × 5 + 50). A score of 85 premorbid IQ or lower was used to define a low premorbid IQ boundary, following DSM and ICD criteria that this IQ cut-off score is the upper boundary for borderline intellectual functioning (1 SD below normal intellectual functioning) and would thus represent a vulnerable group [57]. In addition, previous studies have used this boundary to split samples based on preserved or compromised intellect [51,58]. We explored inter-rater reliability for the Vocabulary test using 10 cases compared with a gold standard score, derived from the consensus of three expert evaluators in the administration and correction of these tests. Interclass correlation coefficients (ICCs) were calculated for each independent evaluator. We only considered for testing trained evaluators with scores higher than the established cut-off point (ICC > 0.80).

The age of onset of psychotic symptoms was defined by the time of appearance of the first positive psychotic symptom. It was evaluated retrospectively at baseline according to reports from the patient and his/her family and clinical reports. We defined early onset of psychosis as the onset of positive psychotic symptoms in individuals younger than 18 years old and adult onset of psychosis as the onset at 18 years or older [19]. As a result, we classified individuals into four subgroups: Group 1 was early-onset with low pIQ (N = 41, 16.1%); Group 2 was adult-onset with low pIQ (N = 70, 27.5%); Group 3 was early-onset with average pIQ (N = 30, 11.8%); and Group 4 was adult-onset with average pIQ (N = 114, 44.7%).

#### 2.2.2. Clinical Assessment

At baseline, we gathered relevant demographical and clinical data for all participants. Duration of untreated psychosis (DUP) was calculated as the time (days) elapsed between the first positive symptom (delusions, hallucinations, or disorganization) recalled and baseline assessment [59]. We used DSM-IV criteria [60] to establish the diagnosis of psychotic disorder or its absence at baseline using the Spanish version of the structured clinical interview for DSM-IV (SCID) I for axis I, mental major disorders [61] for adults (over 18 years), and the Kiddie Schedule for Affective Disorders and Schizophrenia for children and adolescents (until age 18; [62]). At the two-year follow-up, we revised the diagnosis using the correspondent semi-structured interview designed to assess current and past psychopathology. We grouped the patients into three diagnostic categories: (1) schizophrenia spectrum disorders (SSD), which included schizophrenia, schizophreniform, and schizoaffective disorders; (2) affective spectrum disorders (ASD), which included bipolar disorder I and II, and manic and depressive episodes with psychotic symptoms; and (3) other psychoses (OPs), which included brief psychotic disorders, psychoses not otherwise specified, and toxic psychoses. When we treated diagnosis as a dichotomous variable, we grouped ASD and Ops as “non-SSD” patients.

Participants underwent clinical and functional assessment both at baseline and at two-year follow-up. We used the Global Assessment of Functioning Scale (GAF; ≥18 years) [63] or the Children Global Assessment of Functioning Scale (c-GAF; <18 years) [64], respectively, to assess general functioning. Clinical assessments were conducted with the Positive and Negative Symptom Scale (PANSS) [65,66]. Experienced psychiatrists or psychologists administered the assessments and the reliability of the different clinicians administering PANSS scale was evaluated to achieve a within-class correlation coefficient higher than 0.8.

At two-year follow up, we applied Andreasen’s Remission Criteria [67] based on PANSS scores; Andreasen defines “symptom remission” as the presence of scores of = or <3 in the following symptoms of the PANSS: delusions, conceptual disorganization, hallucinations, blunted affect, emotional withdrawal, lack of spontaneity and flow of conversation, mannerisms and posturing, and unusual thought content). The Andreasen’s time criteria was not applied to this definition of remission. We defined a cut-off point of ≥70 in the GAF and c-GAF (range of scores from 1 to 100) for “good functioning” at two-year follow-up, as previous studies have determined [59]. Finally, we defined “clinical recovery” as both good functioning (GAF ≥ 70) and symptom remission at two-year follow-up [68].

We gathered antipsychotic prescription information and converted it into chlorpromazine equivalents based on international consensus [69].

### 2.3. Statistical Analyses

Demographic and clinical characteristics of the sample were analyzed using descriptive statistics (frequencies or mean and SD, as appropriate). The distribution of the continuous variables (demographic and clinical measures) was ascertained using the Kolmogorov–Smirnov and Shapiro–Wilk test. The equality of the variance between subgroups was assessed using Levene’s test. Differences between subgroups were examined using univariate ANOVA for continuous variables and chi-square for categorical variables. To correct for multiple comparisons, Bonferroni and Benjamini Hochberg post-hoc methods were applied [70] and to calculate effect sizes, Cohen’s *d* for post-hoc ANOVA pairwise comparisons (small effect *d* > 0.2, medium effect *d* > 0.5, large effect, *d* > 0.8) and Cramer’s *V* for chi-square pairwise comparisons (small effect *V* > 0.1, medium effect *V* > 0.3, and large effect *V* > 0.5) were used. Secondary comparison analyses through one-way ANCOVA were conducted to explore the potential effects of antipsychotic medication as a confounding variable. Furthermore, to examine differences in clinical and functional trajectories between subgroups, we conducted a two-way mixed ANOVA to assess the effect of group, time and group x time interaction over global functioning (GAF/c-GAF scores), and severity of symptoms (PANSS scores), with partial Eta-Squared (*η_p_*^2^) as a measure of the effect size (small effect *η_p_*^2^ > 0.01, medium effect *η_p_*^2^ >0.06, and large effect *η_p_*^2^ > 0.14).

To examine the predictive value of pIQ and age of onset on global functioning at two-year follow-up (GAF/c-GAF scores) we used a hierarchical multiple regression model, entering pIQ and age of onset in the first block (using the enter method). In the second block, we entered clinical dimensions at baseline that correlated with GAF/c-GAF scores in the bivariate analyses (clinical diagnosis, PANSS subscales scores) using the forward method. The diagnosis variable, SSD or non-SSD, was used as a dichotomous variable with the latter group as the reference category. Furthermore, to test the predictive capacity of pIQ and age of onset for clinical recovery at two-year follow-up, we performed a logistic regression analysis and a multiple logistic regression. Statistical analyses were performed using IBM SPSS Statistic for Windows Version 26.0, IBM Corp., Armonk, NY, USA, 2019.

## 3. Results

### 3.1. Sociodemographic and Clinical Characteristics

The socio-demographic characteristics of the sample are summarized in Table 1. Comparisons between the four first episode of psychosis subgroups revealed no significant differences in socioeconomic status or DUP. Within the adult-onset subgroups, there were significantly more females in the subgroup with low pIQ than in the subgroup with average pIQ. Early-onset with low pIQ subgroup had a lower premorbid adjustment at infancy than adult-onset patients with average pIQ. Regarding diagnosis, both early-onset subgroups had significantly more individuals with a diagnosis of ASD compared to both adult-onset subgroups. Both adult-onset subgroups had significantly more individuals with a diagnosis of OPs than the subgroup of early-onset with average pIQ (see Table 1). Early-onset individuals with average pIQ had a significantly lower main daily dose of antipsychotic treatment at baseline than both adult-onset subgroups. At two-year follow-up, among adult-onset patients, the subgroup with low pIQ had a significantly higher main daily dose of antipsychotic prescription than the subgroup with average pIQ (see Table 1). Within the early-onset age subgroups, we found no differences other than age between adolescents younger than 14 years old and adolescents 14 years old and older (see Supplementary Table S1).

In addition, regarding the sample selected for this study, we found no differences between individuals who completed the required evaluations (n = 255) and those who did not complete them (n = 190) in terms of age (t (443) = 1.46, *p* = 0.14), gender (χ^2^ (1) = 0.002, *p* = 1.00), SES (χ^2^ (4) = 7.61, *p* = 0.11), age at first episode (t (427) = 1.16, *p* =0.25), GAF/c-GAF score at baseline (t (442) = −0.47, *p* = 0.64), PANSS total score at baseline (t (442) = 1.32, *p* = 0.18), or antipsychotic doses at baseline (t (443) = −0.81, *p* = 0.42).

### 3.2. Pairwise Comparison of Clinical Symptoms, Functioning, Symptom Remission, and Clinical Recovery Rates

Comparisons in clinical, functional variables, symptom remission, and clinical recovery rates among first episode of psychosis subgroups are summarized in Table 2.

**Figure 1:**
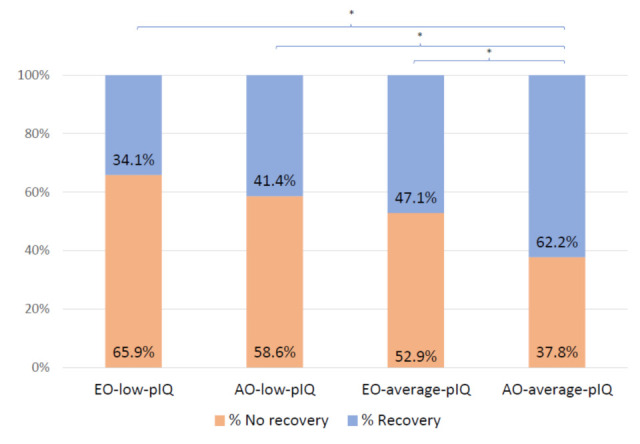
Recovery rates in subgroups of patients with a first episode of psychosis at two-year follow-up. EO-low-pIQ: early onset with premorbid IQ < 85. AO-low-pIQ: adult onset with premorbid IQ < 85. EO-average-pIQ: early onset with premorbid IQ ≥ 85. AO-average-pIQ: adult onset with premorbid IQ ≥ 85. * = Significant pairwise comparison *p* < 0.05.

Within the groups with the same age range at onset (early-onset with pIQ vs. early-onset with average pIQ; adult-onset with low pIQ vs. adult-onset with average pIQ), the only difference in symptomatology, functioning, remission, or recovery rates at baseline or follow-up was that the subgroup of adult-onset with average pIQ recovered significantly more frequently than adult-onset with low pIQ subgroup (*V* = 0.17). We found no significant difference in these clinical variables within the subgroups of early-onset cases. Figure 1 reflects the progression pattern of recovery rates for the four subgroups.

Within the same IQ range (early-onset with low pIQ vs. adult-onset with low pIQ; and early-onset with pIQ vs. adult-onset with average pIQ), both early-onset patients subgroups had significantly more severe positive symptoms and poorer functioning at baseline than both subgroups of adult-onset patients (*d =* 0.51 to 1.21; see Table 2), with higher effect sizes for the pairwise comparison between the subgroups of early-onset with low pIQ and adult-onset with low pIQ (*d* positive symptoms = 1.21; *d* functioning = 1.17). Within average-pIQ patients, early-onset cases had significantly lower symptom remission and clinical recovery rates than adult-onset patients (remission: *V* = 0.16; recovery: *V* = 0.14).

Furthermore, comparisons between the four first episode of psychosis subgroups revealed significant clinical and functional differences between the subgroups of early-onset with low pIQ and adult-onset with average pIQ. Early-onset patients with low pIQ showed higher positive (*d =* 0.78) and total symptoms score (*d =* 0.71) at baseline, higher severity of negative symptoms (*d =* 0.59), and total symptoms score (*d =* 0.5) at two-year follow-up, worse functioning both at baseline (*d =* 1.29) and at follow-up (*d =* 0.82), and lower remission and recovery rates than the subgroup of adult-onset with average pIQ (remission: *V =* 0.21; recovery: *V =* 0.25).

In our secondary analysis, controlling for medication, the only effect that disappeared was the difference between the subgroups of early-onset with low pIQ and adult-onset with average pIQ in PANSS total score at two years (F(3) = 1.99, *p* = 0.12).

### 3.3. Clinical and Functioning Changes over Time

**Figure 2:**
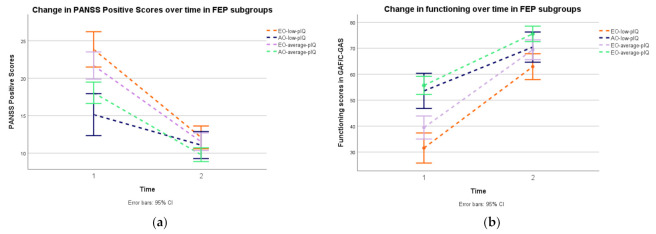
Change in positive symptoms (**a**) and functioning outcome (**b**) in subgroups of patients with a first episode of psychosis from baseline to two-year follow-up. Error bars: 95% CI. EO-low-pIQ: early onset with premorbid IQ < 85; AO-low-pIQ: adult onset with premorbid IQ < 85. EO-average-pIQ: early onset with premorbid IQ ≥ 85. AO-average-pIQ: adult onset with premorbid IQ ≥ 85.

Positive, negative, and total symptomatology improved for the whole sample during follow-up with a corresponding significant main effect of time (positive: F (247, 1) = 194.08, *p* ≤ 0.001, *η_p_*^2^ = 0.44; negative: F (1, 247) = 36.36, *p* < 0.001, *η_p_*^2^ = 0.13; total: F (1, 247) = 163.85, *p* < 0.001 *η_p_*^2^ = 0.40). We found a significant group x time interaction effect for positive symptoms (F (247, 3) = 5.29, *p* = 0.001, *η_p_*^2^ = 0.06), with early-onset subgroups showing a significant larger improvement in positive symptoms scores over time (from baseline to two-year follow-up) than adult-onset subgroups (*p* = 0.007 to *p* < 0.001 in group x time paired comparisons; see Figure 2a). There were no significant changes from baseline to two-year follow-up (within-group differences) in negative symptoms for the subgroups with low pIQ (early-onset-low-pIQ *p* = 0.06; adult-onset-low-pIQ *p* = 0.07). Time x group interaction in negative symptoms (F (1, 247) = 0.26, *p* = 0.85) or total score symptomatology (F (1, 247) = 2.45, *p* = 0.06) variables were not significant.

Figure 2b shows baseline and two-year functioning according to the global assessment functioning scales. We found a significant group x time interaction (F (251, 3) = 4.42, *p =* 0.004, *η_p_*^2^ = 0.06). The within group x time paired comparisons revealed that only the subgroup of early-onset with low pIQ had an improvement trajectory significantly different from the three other subgroups (*p* = 0.04 to *p* < 0.001). Figure 2b show that the subgroup of early-onset with low pIQ subgroup presented with the highest improving in functioning over time but had the lowest GAF score both at baseline (mean= 31.56, SD = 17.26) and at two-year follow-up (mean *=* 62.9, SD *=* 18.68). On the contrary, the subgroup of adult-onset with average pIQ achieved the highest score at the two time-points (mean at baseline *=* 55.58, SD *=* 19.11, mean at the follow-up *=* 75.53, SD *=* 13.31) with the lowest increase trajectory in GAF/c-GAF scores.

### 3.4. Predictive Value of pIQ and Age of Onset at Baseline over General Functioning at Two-Year Follow-Up

Age of onset and pIQ significantly predicted the improvement in GAF/c-GAF scores at two-year follow-up (*F* (2, 252) = 9.936, *p* < 0.001, *R*^2^ adjusted = 0.07), and explained a 7% of the variance of two-year follow-up global functioning scores (both variables added statistically significantly to the prediction, *p* < 0.05; see Supplementary Table S2). The interaction of age of onset and pIQ significantly predicted global functioning (*F* (1, 254) = 19.15, *p* < 0.001, *R*^2^ adjusted = 0.07) without increasing the predictive value of the two variables (age of onset and pIQ).

Among clinical variables, only diagnosis at baseline was significant (*p* < 0.001) and the model explained a total variance of 16% (model 2, F = 16.75, *p*<0.001, *R*^2^ adjusted *=* 0.16; see Supplementary Table S3). Age of onset and pIQ had a positive association with GAF/c-GAF two-year scores, and diagnosis had a negative association. That is to say, diagnosis of affective psychosis or psychosis NOS was related with better GAF/c-GAF two-year scores than diagnosis of schizophrenia spectrum disorder (see Supplementary Table S3).

We used a logistic regression to assess the predictive power of age of onset and pIQ for clinical recovery with the final model explaining 6% (Nagelkerke R2) of the variance in clinical recovery and correctly classifying 55.4% of cases (χ^2^ (2) *=* 11.10, *p* < 0.001). Sensitivity was 69% and specificity was 70%. Of the two predictor variables, only premorbid IQ was statistically significant (as shown in Table S4). Increased pIQ was associated with an increased likelihood of exhibiting clinical recovery. The area under the ROC curve was 0.61 (CI 0.54, 068) [71]. We performed a secondary binomial logistic regression using the forward stepwise method to ascertain the effect of age of onset, pIQ and diagnosis (non-SSD or SSD) on the likelihood that participants would have clinical recovery. The model that improved the prediction of clinical recovery likelihood (Model 2) included the variables pIQ (*p* = 0.013) and diagnosis (*p* < 0.001), was statistically significant (χ^2^ (2) = 41.076, *p* < 0.001) explaining 20% (Nagelkerke *R*^2^) of the variance in clinical recovery and correctly classifying 67.3% of cases. Sensitivity was 76% and specificity was 93% (as shown in Table S5). Higher pIQ and having a non-SSD diagnosis was associated with an increased likelihood of exhibiting clinical recovery. The area under the ROC curve was 0.718 (95%CI 0.655, 0.782) [71].

## 4. Discussion

In this study, we aimed to explore the combined effect of premorbid IQ and age of onset of psychotic symptoms on psychotic symptomatology and functioning over two years of follow-up on a sample of first episode of psychosis. We found that subgroup of early-onset with low pIQ presented with the worst global psychosocial functioning and that, despite the trajectory of improvement over time, this subgroup still showed poor functioning at two-year follow-up. Conversely, the subgroup of adult-onset with average pIQ presented with average functioning at two-year follow-up, with intermediate values for the other two subgroups.

At baseline, early-onset individuals showed more severe symptoms, with higher scores in positive and general subscales and PANSS total score than adult-onset participants. All four subgroups improved on positive symptoms at two-year follow-up with the greatest improvement for early-onset participants, perhaps due to their higher baseline rates (i.e., EO subgroups had the most “room for improvement”). In addition, both average-pIQ subgroups improved on negative symptoms at the follow-up. However, the subgroups with low pIQ did not show significant change of negative symptoms’ severity over time, with only individuals in the subgroup of early-onset with low pIQ showing significantly more negative symptoms than individuals in the adult-onset with average-pIQ group at the follow-up.

Adult-onset with average pIQ was the subgroup that achieved the highest remission rate (81.1%), which was significantly higher than both early-onset subgroups (early-onset-average-pIQ: 67.1%; early-onset-low-pIQ: 61%). In the case of full recovery (both symptomatic and functioning), the subgroup of adult-onset with average pIQ significantly differed from the other three subgroups. More than 60% of adult-onset patients with average pIQ fully recovered, with fewer than 40% in the early-onset with low-pIQ subgroup and intermediate values for the other two subgroups.

Our results suggest heterogeneity in the presentation and clinical and functioning outcomes at two-year follow-up in first-episode psychosis depending on age of onset and pIQ. This is in line with previous works that support the hypothesis of an influence of age of onset on clinical outcomes [18,21,27]. Our findings also suggest that paying attention to the interplay of age of onset and pIQ may help identify early-onset individuals with more compromised neurodevelopment. Among early-onset patients, only the low-pIQ subgroup did not show an improvement of negative symptoms at the follow-up. This suggests the possibility that the profile of negative symptoms differs between both early-onset subgroups. Previous investigators have discussed how negative symptoms within psychosis might comprise different phenomena, including primary negative psychotic symptoms (defectual) but also secondary manifestations derived from medication [30,72]. The presence of poor premorbid cognitive abilities, perhaps in the form of language poverty or concrete thinking, together with low functioning suggest primary negative symptoms in the EO patients with low pIQ. Our results led us to consider negative symptom trajectories as an important factor in early-onset patients which, together with low pIQ constitute factors associated with deviant developmental processes present before the onset of psychotic symptoms [73]. Indeed, the persistence of negative symptoms in this subgroup warrants finer-grain analysis of negative symptoms. One example would be to assess primary negative symptoms with the criteria proposed by Galderisi et al. [74] in order to ascertain if the primary and enduring negative symptoms are present more frequently in those patients with early onset and low pIQ than in the other subgroups, which may show secondary negative symptoms, such as depressive symptoms, due to other factors. Puig et al. [30] compared the prevalence of persistent negative symptoms between early-onset and adult-onset psychosis and found that an early onset of psychosis increased the odds of meeting criteria for primary negative symptoms and that those patients had greater global cognitive deficits. Lack of insight has been a variable with state and trait characteristics (these latter ones associated with schizophrenia and particularly early onset) that could have a role in the persistence of negative symptoms [75,76]. Musket et al. [40] also identified shared genetic effects between age at onset and negative symptom severity in patients with schizophrenia. Additional evidence supports the idea that early onset of schizophrenia is moderately heritable [39,40] and that a familial component exists in the relationship between cognitive performance and psychosis [41,42]. Thus, the presence of an early age of onset and a low pIQ may reflect a specific neurodevelopmental course within psychotic disorders [43].

In this vein, Abdin et al., [77] examined the heterogeneity in trajectories of symptom severity in individuals who have experienced a first episode of psychosis and their impact on functioning, and identified a group of patients associated with younger age, male sex, lower education, longer DUP, and diagnosis of SSD, with higher risk of symptom severity and poor functioning at the two-year follow-up. In our study, both age of onset and pIQ have proven to be predictors of functioning outcomes at two-year follow-up (GAF/C-GAS score). Moreover, both hierarchical linear regression and multiple logistic regression analyses showed that including baseline diagnosis in our model improves our predictions of GAF/c-GAF scores and recovery rates at two-year follow-up. We found that SSD was related to a poorer functioning prognosis. The latter is consistent with abundant previous works which signal that first-episode psychosis individuals with an initial diagnosis of non-affective psychosis were more likely to experience a worse clinical course and global functioning, worse socio-occupational outcomes, and poorer quality of life at follow-up [78,79,80].

In our study, recovery rates ranged from 34.1% to 62.2% at two-year follow-up. In a recent systematic review and meta-analysis from Lally et al. [16], a pooled prevalence of long-term recovery among 9642 individuals with FEP was estimated as 38% (35 studies, mean follow-up 7.2 years). Other works with shorter follow-up periods (1–3 years) showed percentages of patients with global recovery of around 13.5% and 26% [11,81]. Recovery rates, according to clinicians [82] is a useful concept, a clinical priority, and a therapeutic goal [83], given that even if patients reach symptomatic stability, the low functioning of patients requires a re-evaluation of treatment [84]. Differences in recovery rates among studies could be related to not having uniform criteria for recovery [84].

Given our results, it seems useful to consider age at onset and pIQ as variables that contribute to variability in clinical and functioning outcomes and as prognostic factors. However, since in logistic regression only the variable pIQ showed prognostic value, it is worth emphasizing the value of cognitive performance to functional outcomes. Early-onset patients had lower functioning than adult-onset patients at baseline, however, those early-onset patients with average pIQ had a better functioning trajectory than those with low pIQ, which might indicate that preserved pIQ serves as a protective factor for better functional outcomes. Preservation of premorbid cognition has been considered a proxy indicator of cognitive reserve (CR), which may be a protective factor against brain damage and functional impairment [85]. In line with our results, evidence suggests that CR in psychotic patients is associated with clinical and psychosocial functioning improvement [86,87,88,89], and training programs to stimulate intellectual skills are recommended [88]. Based on our results, the implementation of cognitive and functional therapeutics strategies seems especially relevant in the case of adolescents with low pIQ in order to reduce the long-term impact of the illness.

However, our study has several limitations. First, although the original sample size is large (N = 255), the sample sizes for the subgroups are relatively small, except the adult-onset with average pIQ subgroup, which is a finding in itself given the prevalence of lower vs. higher pIQ in the EOP subsample. Second, although the GAF is widely used and recognized as a measure of global functioning, other validated tools such as the Functioning Assessment Short Test, FAST might have been more precise in defining functional dysfunction according to relevant domains other than general functioning [90,91]. Furthermore, we did not have specific clinical scales to assess negative symptomatology, such as the Brief Negative Symptom Scale [92] or the Clinical Assessment Interview for Negative Symptoms [93]. Finally, the relatively short follow-up period of two years could be insufficient for detecting the stabilization of good functional outcomes and symptom remission, so studies using data spanning longer follow-up periods will be valuable in order to replicate, challenge, or extend the results of the present study. The main strength of this study is the large and well-characterized cohort of FEP patients with a wide range of age of onset, including early-onset and adult-onset individuals in a naturalistic and longitudinal study.

## 5. Conclusions

In summary, we have explored heterogeneity among individuals who have experienced a first episode of psychosis based on two factors that have been consistently linked with relevant outcome measures: age at onset and pIQ. Through subgrouping research strategy, we have identified a group of patients with early onset and low pIQ of greater vulnerability by presenting the highest severity of negative symptoms at two-year follow-up, the worst functioning at both the baseline and two-year follow-up, and the lowest recovery rate. Understanding this variability is critical to the development of personalized treatment interventions that take into account age of onset and premorbid intellectual performance to improve functional recovery. This should also motivate further exploration of the neuropsychological performance in these FEP subgroups in order to characterize whether they have broad neurocognitive impairments or domain-specific deficits, the relationship between cognitive performance and functioning, and the possible link with neurobiological substrates that allow us to better understand the disease etiology.

## Figures and Tables

**Table 1:** Demographic characteristics of the whole sample of individuals who have experienced a first episode of psychosis and by subgroups classified as: early-onset with low-pIQ, adult-onset with low-pIQ, early-onset with average-pIQ, early-onset with average-pIQ.

		1	2	3	4				
Clinical Ratings	Whole FEP Sample	Early Onset	Adult Onset	Early Onset	Adult Onset				
		Low pIQ		Average pIQ					
mean (SD) [95% IC]	*N* = 255	*N* = 41	*N* = 30	*N* = 70	*N* = 114	Statistic		Significant Post-Hoc Comparison	
						F/χ^2^ (*d.f.*)	Sig. *(p)*	Pair Comparisons	*p*
Age of symptoms onset	21.31 (6.03) [20.56–22.05]	15.9 (1.78) [15.34–16.47]	23.31 (4.60) [21.60–25.03]	16.18 (1.55) [15.81–16.55]	25.87 (4.96) [24.95–26.80]				
Baseline age	21.66 (6.06) [20.91–22.41]	16.17 (1.77) [15.61–16.73]	23.80 (4.69) [22.05–25.56]	16.48 (1.59) [16.10–16.86]	26.25 (4.93) [25.34–27.17]				
Estimated premorbid IQ	91.65 (15.31) [89.76–93.54]	71.59 (7.19) [69.31–73.86]	74.83 (6.22) [72.51–77.16]	97.36 (11.51) [94.61–100.1]	99.78 (10.51) [97.83–101.73]				
Sex *N* (%) Female	83 (32.5)	14 (34.1)	16 (53.33)	23 (32.9)	30 (26.3)	7.97	0.047 ^b^	2 > 4	0.005
Parental SES–*N* (%)						13.76 (12)	0.32^b^		
High	48 (19.0)	7 (17.1)	4 (13.3)	11 (16.2)	26 (22.8)				
Medium High	31 (12.3)	6 (14.6)	1 (3.3)	9 (13.2)	15 (13.2)				
Medium	62 (24.5)	4 (9.8)	9 (30.0)	19 (27.9)	30 (26.3)				
Medium Low	79 (31.2)	17 (41.5)	11 (36.7)	18 (26.5)	33 (28.9)				
Low	33 (13.0)	7 (17.1)	5 (16.7)	11 (16.2)	10 (8.8)				
DUP, mean (SD) [range]	129.79 (124.61) [114.30–145.28]	95.32 (109.49) [60–76–129.87]	180.69 (114.44) [137.15–224.21]	111.14 (11.31) [84.40–137.89]	140.72 (135.30) [115.39–166.06]	3.57 (3)	0.015 ^a^	2 > 1	0.027
PAS Infancy	0.73 (0.18) [0.71–0.75]	0.67 (0.2) [0.61–0.74]	0.69 (0.22) [0.60–0.78]	0.72 (0.19) [0.68–0.77]	0.77 (0.15) [0.73–0.79]	3.14 (3)	0.026 ^a^	1 < 4	0.04
Baseline AP main daily dose	522.30 (401.08) [181.08–246.67]	505.37 (527.58) [338.84–671.88]	681.42 (421.14) [518.12–844.72]	348.47 (192.72) [302.17–394.76]	596.46 (404.87) [520.30–672.62]	7.65 (3)	<0.001 ^a^	3 < 2 3 < 4	0.001 <0.001
Two-year AP main daily dose	0.73 (0.18) [0.71–0.75]	280.58 (291.81) [184.66–376.50]	298.86 (330.56) [176.01–301.07]	238.54 (252.34) [176.01–301.07]	154.69 (221.36) [113.44–195.95]	4.17 (3)	0.007 ^a^	4 < 2	0.04
Baseline diagnosis *N* (%)						22.58 (6)	0.001 ^b^	ASD 1 > ASD 2 ASD 1 > ASD 4 ASD 3 > ASD 2 ASD 3 > ASD 4 OPs 3 < OPs 2 OPs 3 < OPs 4	0.004 0.02 <0.001 <0.001 0.001 0.002
Schizophrenia Spectrum Disorders (SSD)	150 (58.8%)	25 (61.0%)	17 (56.7%)	45 (64.3%)	63 (55.3%)				
Affective Spectrum Disorders (ASD)	51 (20%)	11 (26.8%)	2 (6.7%)	20 (28.6%)	18 (15.8%)				
Other Psychoses (OPs)	54 (21.2%)	5 (12.2%)	11 (36.7%)	5 (7.1%)	33 (28.9%)				

Significance at *p* < 0.05. Abbreviations: FEP = first episode psychosis; low-pIQ = premorbid IQ < 85; average pIQ = premorbid IQ ≥ 85; SES = parental socio-economic status using Hollingshead’s Two-Factor Index of Social Position (Hollingshead and Redlich, 1958). DUP = duration of untreated psychosis. DUP was calculated as the number of days between the first manifestation of psychotic symptoms and the initiation of the treatment. Premorbid Adjustment Scale (Cannon-Spoor, Potkin, and Wyatt, 1982) based on information from patients and parents or close relative. Diagnosis was assessed at baseline and reviewed at two years. SSD = schizophrenia spectrum disorders, which include: schizophrenia, schizophreniform, and schizoaffective disorders; ASD = affective spectrum disorders, which included: bipolar disorder I and II, and manic and depressive episodes with psychotic symptoms; Ops = other psychoses, which include: brief psychotic disorders, psychoses not otherwise specified, and toxic psychoses. ^a^ = ANOVA pair-wise comparison corrected with Bonferroni. ^b^ = the significance of χ^2^ for multiples comparisons was corrected with the Benjamini Hochberg method.

**Table 2:** Clinical and functional characteristics of the sample of individuals who have experienced a first episode of psychosis and differences among subgroups classified as: early-onset with low-pIQ, adult-onset with low-pIQ, early-onset with average-pIQ, adult-onset with average-pIQ.

Clinical Ratings	Whole FEP Sample	1	2	3	4					
		Early Onset	Adult Onset	Early Onset	Adult Onset					
		Low pIQ		Average pIQ						
	*N=* 255	*N=* 41	*N=* 30	*N=* 70	*N=* 114	Test Statistic		Significant *post-hoc* Comparison		
						F/χ^2^ *(d.f.)*	Sig. *(p)*	Pair Comparisons	*p*	*d/V*
**PANSS Positive Symptoms baseline mean** (SD) [95% CI]	19.54 (8.12) [18.09–20.46]	23.85 (6.57) [21.77–25.93]	15.23 (7.18) [12.55–17.91]	21.60 (7.69) [19.76–23.43]	17.86 (8.14) [16.34–19.37]	10.96 (3)	<0.001 ^a^	1 > 2	<0.001	1.21
								1 > 4	<0.001	0.78
								3 > 2	0.001	0.29
								3> 4	0.009	0.51
**PANSS Positive Symptomsmean at two years** (SD) [95% CI]	10.79 (5.00) [9.78–11.11]	12.10 (6.28) [10.11–14.08]	11.07 (5.56) [8.95–13.18]	11.52 (5.47) [10.20–12.83]	9.79 (3.73) [9.08–10.48]	3.38 (3)	0.03 ^a^	n.s.		
**PANSS Negative Symptoms baselinemean** (SD) [95% CI]	18.67 (8.52) [17.03–19.31]	20.63 (10.72) [17.24–24.01]	19.03 (9.05) [15.65–22.41]	19.03 (8.84) [16.92–21.13]	17.65 (7.15) [16.32–18.97]	1.44 (3)	0.26 ^a^	n.s.		
**PANSS Negative Symptomsmean at two years** (SD) [95% CI]	14.73 (6.84) [13.02–14.89]	17.49 (8.18) [14.90–20.07]	15.62 (6.82) [13.02–18.21]	14.84 (7.29) [13.08–16.59]	13.42 (5.69) [12.35–14.48]	4.18 (3)	0.01 ^a^	1 > 4	0.005	0.59
**PANSS General Symptoms baselinemean** (SD) [95% CI]	39.09 (13.24) [36.63–40.28]	44.83 (14.90) [40.12–49.53]	35.10 (13.10) [30.20–39.99]	41.03 (13.41) [37.83–44.22]	36.89 (11.76) [34.70–39.06]	5.27 (3)	0.002 ^a^	1 > 2 1 > 4	0.01 0.005	0.60 0.61
**PANSS General Symptoms mean at two years** (SD) [95% CI]	43.04 (18.45) [40.17–45.31]	37.02 (19.15) [30.97–43.07]	53.45 (19.25) [46.12–60.77]	34.07 (15.81) [30.30–37.84]	48.16 (16.44) [45.08–51.23]	15.21 (3)	<0.001 ^a^	1 < 2	0.001	0.93
								1 < 4	0.003	1.27
								3 < 2	<0.001	0.67
								3 < 4	<0.001	0.92
**PANSS Total baselinemean** (SD) [95% CI]	77.30 (25.17) [72.43–79.40]	89.32 (26.45) [80.96–97.66]	69.37 (26.07) [59.63–79.10]	81.66 (24.77) [75.74–87.56]	72.39 (22.86) [68.15–76.63]	6.63 (3)	<0.001 ^a^	1 > 2 1 > 4	0.005 0.001	0.68 0.71
**PANSS Totalmean at two years** (SD) [95% CI]	51.83 (19.06) [47.56–52.79]	57.98 (23.04) [50.70–65.24]	53.45 (19.25) [46.12–60.77]	53.16 (19.94) [48.36–57.95]	48.34 (16.19) [45.31–51.37]	3.17 (3)	0.035 ^a^	1 > 4	0.026	0.5
**GAF baselinemean** (SD) [95% CI]	47.10 (21.08) [46.49–52.52]	31.56 (17.26) [26.11–37.01]	53.57 (18.52) [46.65–60.48]	39.46 (19.46) [34.81–44.09]	55.68 (19.11) [52.13–59.22]	22.19(3)	<0.001 ^a^	1 < 2	<0.001	1.17
								1 < 4	<0.001	1.29
								3 < 2	0.004	0.72
								3 < 4	<0.001	0.88
**GAFmean at two years** (SD) [95% CI]	71.22 (16.67) [70.85–75.53]	62.90 (18.68) [57.01–68.79]	70.43 (14.03) [65.19–75.67]	69.40 (19.32) [64.79–74.01]	75.53 (13.31) [73.05–77.99]	6.51 (3)	<0.001 ^a^	1 < 4	<0.001	0.82
**Good functioning at two years *N*** (%)	135 (52.9)	14 (34.1)	14 (46.7)	35 (50)	72 (63.2)	11.31 (3)	0.01 ^b^	1 < 4	0.001	0.26
**Symptom remission at two years *N*** (%)	181 (71)	25 (61)	19 (65.5)	47 (67.1)	90 (81.1)	8.45(3)	0.03 ^b^	1 < 4	0.01	0.21 0.16
								3 < 4	0.03	
**Recovery at two years *N*** (%)	128 (51)	14 (34.1)	12 (41.4)	33 (47.1)	69 (62.2)	11.69(3)	0.009 ^b^	1 < 4	0.002	0.25 0.17 0.14
								2 < 4	0.04	
								3 < 4	0.04	

Significance at *p* < 0.05. Abbreviations: FEP: first-episode psychosis; low-pIQ = premorbid IQ < 85; average-pIQ = premorbid IQ ≥ 85; ^a^ = ANOVA pair-wise comparison corrected with Bonferroni; ^b^ = the significance of χ^2^ for multiples comparisons was corrected with the Benjamini Hochberg method. Bold values indicate significance at *p* < 0.05 and size effect medium-large. Cohen’s d was calculated as an effect size estimate for post-hoc ANOVA pairwise comparisons (small effect d > 0.2, medium effect d > 0.5, large effect, d > 0.8) and Cramer’s V for χ^2^ (small effect *V* > 0.1, medium effect *V* > 0.3, large effect, *V* > 0.5). Symptom remission: defined as the presence of scores of = or <3 in the following symptoms of the PANSS: delusions, conceptual disorganization, hallucinations, blunted affect, emotional withdrawal, lack of spontaneity and flow of conversation, mannerisms and posturing, and unusual thought content. Good functioning: ≥70 in the GAF and CGAF (range of scores from 1 to 100). Clinical recovery: defined as the presence of both good functioning (GAF ≥ 70) and symptom remission at two-year follow-up.

## Supplementary Materials

The following are available online at https://www.mdpi.com/article/10.3390/jcm10112474/s1, Table S1. Demographic and clinical characteristics of the participants with early-onset of psychosis classified as: younger than 14 years old and adolescents 14 years and older. Table S2: Multiple linear regression model assessing the association of age of onset and premorbid IQ with GAF/c-GAF at two-year follow-up; Table S3: Multiple regression predicting GAF/c-GAF at two-year follow-up from age of onset, premorbid IQ interaction, and diagnosis in the whole sample of first episode of psychosis patients; Table S4: Logistic regression predicting likelihood of heart disease based on age of onset and premorbid IQ; and Table S5: Logistic regression predicting GAF/c-GAF at two-year follow-up from age of onset, premorbid IQ and diagnosis in the whole sample of first episode of psychosis patients.
