# Supplementary material for: The Role of Premorbid IQ and Age of Onset as Useful Predictors of Clinical, Functional Outcomes, and Recovery of Individuals with a First Episode of Psychosis
Source: J Clin Med. 2021 Jun 2;10(11):2474. doi: 10.3390/jcm10112474 (PMC8199787; doi:10.3390/jcm10112474)
Supplement: Supplementary file 1 [file jcm-10-02474-s001.zip › Molina-Garc¿¬a_et_al._jcm-1233120_supplementary_tables_reviewed 04.06.2021.pdf]

SUPPLEMENTARY TABLES

Table S1.
Demographic and clinical characteristics of the participants with early-onset of psychosis classified as: younger than 14 years old and adolescents 14 years and older.

	Participants <14	*Participants ≥14 - <18*		
	N=9	N=96	Statistic	
			**T / X^2** *(d.f.)*	**Sig.***(p)*
**Age of symptoms onset mean, SD**	12,037 ±1,50	16,359 ±1,07	-11,175 (103)	0,000
**Baseline age mean, SD**	12,27 ±1,5	16,63 ±1,04	-11,590 (103)	0,000
**Estimated premorbid IQ mean, SD**	86,11 ±18,67	86,82 ±15,24	-0,131 (103)	0,896
**Sex- N(%)**<br>**Female**<br>**Male**	3 (33,3)<br>6 (66.7)	33 (34.4)<br>63 (65.7)	0.004 (1)	1.00
**Parental SES– N(%)**<br>**High**<br>**Medium High**<br>**Medium**<br>**Medium Low**<br>**Low**	2 (22,2)<br>1 (11.1)<br>2 (22,2)<br>3 (33.3)<br>1 (11.1)	14 (14.7)<br>14 (14.7)<br>20 (21.1)<br>31 (32.6)<br>16 (16.8)	0.55 (4)	0.98
**DUP, mean, SD**	84,6667 ±45,94	96,8316 ±104,93	-0,343 (102)	0,732
**Baseline AP main daily dose, mean, SD**	263,8889 ±220,48	431,7829 ±374,33	-1,32 (102)	0,190
**2-year AP main daily dose, mean, SD**	311,43 ±231,84	254,25 ±272,23	0,540 (96)	0,590
**Diagnosis baseline- N (%)**<br>**Schizophrenia spectrum Disorders (SSD)**<br>**Affective spectrum disorders (ASD)**<br>**Other psychosis (OPs)**	8 (88.99)<br>0 (0.0)<br>1 (11.1)	59 (61.5)<br>28 (29.2)<br>9 (9.4)	3.62 (2)	0.16
**PANSS Positive Symptoms baseline mean, SD**	21,44 ±5,74	23,14 ±7,21	-0,683 (103)	0,496
**PANSS Positive Symptoms 2-years mean (SD)**	12,00 ±4,77	11,83 ±5,93	0,082 (103)	0,935
**PANSS Negative Symptoms baseline mean, SD**	20,78 ±8,97	19,48 ±9,72	0,386 (103)	0,701
**PANSS Negative Symptoms 2-years mean, SD**	17,33 ±10,82	15,94 ±7,48	0,514 (103)	0,609
**PANSS General Symptoms baseline mean, SD**	40,11 ±4,96	43,45 ±14,38	-0,690 (103)	0,492
**PANSS General Symptoms 2-years mean, SD**	33,33 ±14,40	35,34 ±17,81	-0,328 (103)	0,743
**PANSS Total baseline mean, SD**	82,33 ±12,25	86,06 ±26,05	-0,424 (103)	0,673
**PANSS Total 2- years mean, SD**	62,67 ±26,80	54,92 ±20,81	1,042 (103)	0,300
**GAF baseline mean, SD**	40,00 ±18,88	34,89 ±18,30	0,800 (103)	0,426
**GAF 2- years mean, SD**	56,78 ±14,57	67,51 ±19,78	-1,585 (103)	0,116

**Table S2.** *Multiple linear regression model assessing the association of age of onset and premorbid IQ with GAF/c-GAF at 2-year follow-up*							
GAF/c-GAF 2-years	*B* (SE)	95% CI for B		β	R^2	ΔR^2	Sig.
		LL	UL				
Model					0.07	0.07	<0.001
Constant	45.47 (6.40)	32.87	58.06				
Age of onset	0.51 (0.18)	0.16	0.86	0.19			0.004
pIQ	0.16 (0.07)	0.03	0.30	0.15			0.02
pIQ: premorbid IQ. AO: age of onset. GAF/c-GAF: global assessment functioning scale or children global assessment functioning scale.							

**Table S3.** *Multiple regression predicting GAF/c-GAF at 2-year follow-up from Age of Onset, premorbid IQ interaction, and diagnosis in the whole sample of first episode of psychosis patients*								
Variable	Model 1				Model 2			
	B (SE)	β	r^2	Sig.	B (SE)	β	r^2	Sig.
Constant	45.47 (6.40)			<0.001	54.86 (6.32)			<0.001
AO	0.51 (0.18)	0.19	0.18	0.004	0.47 (0.17)	0.17	0.17	0.006
pIQ	0.16 (0.07)	0.15	0.15	0.02	0.14 (0.06)	0.13	0.13	0.04
Diagnosis (non SSD/ SSD)					-10.41 (1.96)	-0.30	-0.32	<0.001
r^2 value	0.07				0.17			
Δr^2	0.07				0.16			
F value	9.93				16.75			
p value	<0.001				<0.001			
pIQ: premorbid IQ. AO: age of onset. GAF/c-GAF: global assessment functioning scale or children global assessment functioning scale. Non-SSD: non schizophrenia spectrum disorder, that is, affective spectrum psychosis and other psychosis. Reference group for diagnosis: non-SSD.								

**Table S4** *Logistic regression predicting likelihood of recovery at 2-year follow-up from age of onset and premorbid IQ*									
	B	SE	Wald	*df*	Odds Ratio	95% CI for Odds Ratio		R^2 Nagelkerke	*p*
						Lower	Upper		
Model								0.058	0.004
Age of onset	0.04	0.02	2.94	1	1.04	0.994	1.09		0.086
Premorbid IQ	0.02	0.01	4.77	1	1.02	1.00	1.0.3		0.029
Constant	-2.59	0.84	9.38	1	0.08				0.002

Table S5.
Logistic regression predicting recovery at 2-year follow-up from Age of Onset, premorbid IQ and diagnosis in the whole sample of first episode of psychosis patients

Variable	Model 1				Model 2			
	B (SE)	Wald	*df*	*p*	B (SE)	Wald	*df*	*p*
Constant	1.008 (0.23)	20.11	1	<0.001	-1.14 (0.88)	1.68	1	0.19
Diagnosis (non SSD/ SSD)	-1.58 (0.28)	31.55	1	<0.001	-1.57 (0.29)	30.17	1	<0.001
pIQ					0.23 (0.009)	6.24	1	0.013
R^2 Nagelkerke	0.172				0.201			
Chi-square	34.55				41.08			
p value	<0.001				<0.001			

pIQ: premorbid IQ. AO: age of onset. SSD: schizophrenia spectrum disorder. Non-SSD: non schizophrenia spectrum disorder, that is, affective spectrum psychosis and other psychosis. Reference group for diagnosis: non-SSD.
